# Health literacy as a moderator of health-related quality of life responses to chronic disease among Chinese rural women

**DOI:** 10.1186/s12905-015-0190-5

**Published:** 2015-04-15

**Authors:** Cuili Wang, Robert L Kane, Dongjuan Xu, Qingyue Meng

**Affiliations:** Shandong University School of Nursing, Jinan, 250012 China; University of Minnesota School of Public Health, Minneapolis, 55455 US; Peking University China Center for Health Development Studies, Beijing, 100191 China

**Keywords:** China, Health-related quality of life, EQ-5D, Health literacy, Chronic disease

## Abstract

**Background:**

Chronic disease is the leading global health threat and impairs patients’ health-related quality of life (HRQoL). Low health literacy is linked with chronic diseases prevalence and poor HRQoL. However, the interaction of health literacy with chronic disease on HRQoL remains unknown. Therefore, we examined how health literacy might modify the association between chronic disease and their HRQoL impacts.

**Methods:**

We conducted a health survey of 913 poor rural women aged 23–57 years in Northwestern China. We assessed health literacy and HRQol using the revised Chinese Adult Health Literacy Questionnaire (R-CAHLQ) and Euroqol-5D (EQ-5D), respectively. Low health literacy was indicated by a cut-off of less than the mean of the factor score. Self-reported preexisting physician-diagnosed chronic disease and socio-demographic characteristics were also included. We fitted log-binomial regression models for each dimension of EQ-5D to examine its association with health literacy and chronic disease. We also ran linear regression models for EQ VAS scores and utility scores.

**Results:**

The low health literacy group was 1.33 times more likely to have a chronic disease than the high health literacy group. Pain/discomfort was the most prevalent impairment, and was more common in the low health literacy group (PR [prevalence ratio] = 1.23; 95% CI = 1.01, 1.50). Chronic disease strongly predicted impairments in all the EQ-5D dimensions, with PRs ranging from 2.14 to 4.07. The association between chronic disease and pain/discomfort varied by health literacy level (health literacy × chronic disease: *P* = 0.033), and was less pronounced in the low health literacy group (PR = 2.15; 95% CI = 1.76, 2.64) than in the high health literacy group (PR = 3.19; 95% CI = 2.52, 4.05). The low health literacy group had lower VAS scores and utility scores, and slightly less decrement of VAS scores and utility scores associated with chronic disease.

**Conclusions:**

Health literacy modified the impacts of chronic disease on HRQoL, and low health literacy group reported less HRQoL impacts related to chronic disease. Research should address health literacy issues as well as root causes of health disparities for vulnerable populations.

## Background

Chronic disease is the leading global health threat, contributing to 63% (36 million) of the 57 million deaths in 2008 [1]. The costs of chronic disease are steep not only for individual, but also for families and society. Chronic disease can seriously impair patients’ health-related quality of life (HRQoL) [2]. HRQoL refers to how individuals subjectively assess their own well-being and ability to physical, psychological, and social functions [3]. As the disease burden from chronic disease rises, more attention is being paid to HRQoL. HRQoL affects both entry and subsequent use of health care and the costs of care [4-7]. However, the HRQoL impacts reported by patients do not always reflect clinical factors [5]; the extent to which self-reported HRQoL can accurately capture individuals’ experiences or conditions may be affected by individual and social factors, as well as quality of care [5,8]. Some population subgroups with minority status, financial deprivation, literacy and language difficulties are particularly vulnerable to receive suboptimal health care and achieve poor health outcomes because of ineffective communication with clinicians or increased barriers to care [9].

Health literacy, “the degree to which individuals have the capacity to obtain, process and understand basic health information and services to make appropriate health decisions”, has been suggested as a realistic approach for engaging individuals in their own medical care and health improvement [10,11]. Unlike fixed demographic characteristics and social structures, which cannot be altered without massive social and political action, health literacy is modifiable and can be strengthened by health education [12]. Health literacy has been associated with a range of health outcomes, such as adherence to medication and other health advice, participation in health and screening programs, self-management of chronic disease, and mortality [10,13-17]. Low health literacy is associated with high prevalence of chronic disease and poor HRQoL [15,18-20].

Not yet examined, however, is the interaction of health literacy with chronic disease on HRQoL. According to Wilson and Cleary’s theoretical model of patient outcomes, individual or social characteristics modify the influence of objective clinical condition on subjective HRQoL [5]. Given that health literacy is a shared function of social and individual factors [10] and may directly affect how one perceives and acts on health concerns [21], health literacy may affect subjective HRQoL responses to objective clinical diagnoses of chronic disease. This study investigates how health literacy may modify the association between chronic disease and its HRQoL impacts in poor rural Chinese women. We hypothesized that individuals with low health literacy would report fewer HRQoL impacts related to chronic disease.

## Methods

### Sample

The study relied on in-person interview data from the Ningxia Women Health Project, a study exploring the association of health literacy with health outcomes among rural women in a poor minority area of Northwestern China. The initial recruitment and data collection for this study have been previously described in detail [22]. Briefly, seven elementary schools were randomly selected from three counties in the minority area and trained investigators received referrals for families of all students in the schools. A total of 913 mothers participated in an in-person structured interview, yielding a response rate of 81.2%. The study was approved by the Institutional Review Board from Shandong University. All the participants provided written consent. The investigators had read the information in the informed consent forms to the participants without formal education in plain language. The participants had been given an opportunity to ask questions and all of their questions had been answered to their satisfaction. Their literate relatives (such as their husbands) signed the informed consent forms on their behalf.

### Measures

Socio-demographic characteristics include age in years, ethnicity, education, income, and geographical location. Participants’ ethnicity was dichotomous (Han versus the Hui minorities). We trichotomized the education variable (no formal schooling, elementary school, and middle school or higher) because nearly half of the sample had no formal education. The income variable was dichotomized as above or below the median annual household per capita disposable income in the surveyed area, which was 2962 yuan ($434US). Participants’ self-reported geographical location was trichotomized as the mountain area, the plain area, and the edge of mountain and plain area. The harshest natural and living environment is the mountain area, with poorer infrastructure and more deficient arable soil and water resources.

Participants were asked whether they had ever been diagnosed by a physician with chronic diseases or conditions. If they answered yes, additional questions about time of diagnosis, treatments, and visits to a physician were asked. The diagnoses and morbidity data were coded according to the Disease Classification of the Fourth NHSS [23]. Twelve chronic conditions were extracted from the codes on the basis of prevalence, disease burden and the reliability of self-report diagnostic classification. Participants were then classified as the presence of chronic disease if they reported at least one of these chronic diseases. Given the fairly young sample, the study focused on the broad dichotomous variable of chronic disease (with and without chronic disease), but did not address specific diseases.

Health literacy was assessed using the revised Chinese Adult Health Literacy Questionnaire (R-CAHLQ) [22,24]. This instrument is a 16-item scale with four domains: health-related knowledge (five items), presenting health information skills (four items), health beliefs (four items) and health behaviors (three items) [22]. Responses are recorded on a 2-point scale (0 = wrong, 1 = correct), with a higher score demonstrating a higher level of health literacy. The instrument is valid for measuring health literacy in the surveyed Chinese population, with a Content Validity Index (CVI) of 0.91 which was computed using ratings of item relevance by 6 experts in health education [25]. The instrument has good internal consistency reliability in this study, with a Cronbach’s alpha coefficient of 0.71. Norms have not been established to indicate a score for “adequate health literacy.” As in a previous investigation, we relied on a cut-off of less than the mean of the factor score to indicate “low” health literacy [22]. Using this threshold, about half of the participants were classified as “low” health literacy, which was in coincidence with the fact that nearly half of the participants had no formal education. Additionally, the health literacy factor score included the weighting factors against dimensions, which may accurately reflect the level of health literacy.

HRQoL was assessed using the European Quality of Life-5 Dimensions (EQ-5D), a standardized generic scale that has been translated into more than 150 languages [26,27]. EQ-5D has been widely used to measure health outcome for its brevity and simplicity, especially in large-scale face-to-face health surveys [2,28]. EQ-5D has been used in the National Health Service Survey in China [29], with acceptable discriminate validity and convergent validity [29,30]. The instrument has good reliability in this study, with a Cronbach’s alpha coefficient of 0.75. EQ-5D consists of five dimensions (mobility, self-care, usual activities, pain/discomfort, and anxiety/depression). The responses record three levels of severity (no impairments, moderate impairments, and extreme impairments) within each EQ-5D dimension. They were used as dichotomous variables (no impairment versus any impairment) in this study. EQ-5D has a visual analog scale (VAS) component, allowing respondents to evaluate their current health status on a range from 0 (worst imaginable health status) to 100 (best imaginable health status). In addition, EQ-5D has been calculated as an aggregated utility index on the basis of the health state value sets and used in economic and clinical evaluation in some countries, such as UK [31]. The value set for the instrument has also been established in China recently [32]. We assessed the preference-based utility score using the value set for China.

### Statistical analyses

We compared participant characteristics by health literacy level using *Pearson Chi-Square* or *t* tests for categorical and continuous variables, respectively. Next, we used log-binomial regression models for each dimension of EQ-5D to determine the adjusted associations (adjusted prevalence ratios, PRs) of health literacy and chronic disease with HRQoL. Socio-demographic variables except education which is highly correlated with health literacy, education, and chronic disease were sequentially entered into the models to examine their relationships with HRQoL, respectively. Then, health literacy-stratified log-binomial regression models were used to obtain adjusted PRs of chronic disease within either low health literacy or high health literacy group. We also conducted separate analyses of those with and without chronic disease to obtain adjusted PRs of health literacy within the two subgroups. The statistical significance of interaction term of health literacy with chronic disease for the entire sample was assessed with *wald χ*^*2*^ tests to determine whether health literacy modified the HRQOL responses to chronic disease. Lastly, we conducted linear regression models predicting EQ VAS scores and utility scores for the entire sample, the health literacy-stratified subsamples, as well as subsamples with and without chronic disease. The statistical significance of interaction term of health literacy with chronic disease for the entire sample was assessed with *t* tests. All data were analyzed with the Stata v.12 (StataCorp, College Station, TX).

## Results

Participants had a mean age of 35 (range 23 – 57) years; approximately 58% were Hui minorities, 47% had no formal education, and 24% had a middle school or higher diploma. Half lived in the plain area and 30% in the mountain area. About 38% of women had chronic diseases: 29% had only one disease, 7% had two, and 2% had three or more diseases. The prevalence of chronic disease did not vary greatly by age (36% for less than 35 years versus 41% for more than 35 years) (*χ*^*2*^ = 2.72, *P* > 0.05); but it differed considerably by educational level (*χ*^*2*^ = 18.68, *P* < 0.001), 27%, 37% and 45%, respectively for middle school or higher, elementary school, and no formal schooling. Nearly 47% of women reported no impairments on all five EQ dimensions. The mean EQ VAS score and utility score were 73 (SD = 18) and 0.88 (SD = 0.16), respectively. Participants’ characteristics by health literacy level are shown in Table 1. The mean of health literacy was 9 (SD = 4), and half the participants reported low health literacy level. Low health literacy was more prevalent among older participants, Hui minorities, those with less education and lower income, residents of mountain areas, and those with a chronic disease. Compared with high health literacy women, low health literacy women reported a higher proportion of impairments in mobility and pain/discomfort dimensions of EQ-5D, low EQ VAS scores and utility scores.

**Table 1:** **Participant characteristics, the prevalence of EQ-5D impairments, VAS and utility scores, by HL** ^**1**^ **level (N = 913)**

	**HL**		
	**Low HL**	**High HL**	***P*** ^**2**^
**N (%)**	463 (51)	450 (49)	
**Age, mean (SD)**	36.2 (5.0)	**34.2 (4.2)**	< 0.001
**Ethnicity: N (%)**			< 0.001
Hui minority	318 (69)	**206 (46)**	
Han	145 (31)	**244 (54)**	
**Education: N (%)**			< 0.001
No formal schooling	351 (76)	**81 (18)**	
Elementary school	88 (19)	**171 (38)**	
Middle school or higher	24 (5)	**198 (44)**	
**Income: N (%)**			< 0.001
Poverty	273 (59)	**171 (38)**	
Non-poverty	190 (41)	**279 (62)**	
**Geographical location: N (%)**			< 0.001
Mountain area	167 (36)	**103 (23)**	
Plain area	213 (46)	**257 (57)**	
The edge of mountain and plain	83 (18)	**90 (20)**	
**Having a chronic disease: N (%)**	203 (44)	**147 (33)**	< 0.001
**EQ-VAS scores**	70.6 (19.1)	**75.5 (16.4)**	< 0.001
**Utility scores**	0.86 (0.17)	**0.89 (0.14)**	< 0.001
**EQ-5D: any impairment: N (%)**			
Mobility	56 (12)	**23 (5)**	< 0.001
Self-care	28 (6)	23 (5)	0.362
Usual activities	69 (15)	68 (15)	0.953
Pain/discomfort	222 (48)	**167 (37)**	< 0.001
Anxiety/depression	162 (35)	135 (30)	0.121

1: HL = health literacy.

2: *Pearson chi-square* (categorical) and *t* (continuous) tests by the HL level. Significant differences were shown in bold.

The adjusted PRs and 95% CI for EQ-5D impairments for the entire sample are shown in Table 2. The PRs for the low health literacy group show higher prevalence of the HRQoL impairment, compared to the high health literacy group. After adjusting for socio-demographics except education (Model 1 in Table 2), the PRs for impairments in mobility and pain/discomfort for women with low health literacy were 1.91 (95% CI = 1.15, 3.16) and 1.24 (95% CI = 1.01, 1.54), respectively. After further adding education (Model 2 in Table 2), the PR for impairments in pain/discomfort (PR = 1.23; 95% CI = 1.01, 1.50) was slightly decreased, and that for impairments in mobility was not statistically significant at the 0.05 level, but borderline significant (PR = 1.74; 95% CI = 0.97, 3.13; *P* = 0.06). However, further inclusion of chronic disease eliminated their associations (Model 3 in Table 2). Chronic disease significantly predicted impairments in all the EQ-5D dimensions, with PRs ranging from 2.14 to 4.07.

**Table 2:** **Adjusted** ^**1**^ **prevalence ratios (PRs) and 95% CI for EQ-5D impairments for the entire sample**

**EQ-5D Impairments**	**Model 1**	**Model 2**	**Model 3**
	**PRs (95% CI)**	**PRs (95% CI)**	**PRs (95% CI)**
**Mobility**			
Low HL^2^ (ref. High HL)	**1.91 (1.15, 3.16)**	1.74 (0.97, 3.13)	1.67 (0.94, 2.98)
With CD^**2**^ (ref. without CD)	N/A	N/A	**3.69 (2.29, 5.96)**
**Self-care**			
Low HL (ref. High HL)	0.95 (0.52, 1.72)	0.84 (0.42, 1.69)	0.81 (0.41, 1.59)
With CD (ref. without CD)	N/A	N/A	**4.07 (2.21, 7.49)**
**Usual activity**			
Low HL (ref. High HL)	0.88 (0.63, 1.23)	1.0 (0.66, 1.51)	0.96 (0.64, 1.43)
With CD (ref. without CD)	N/A	N/A	**2.43 (1.76, 3.34)**
**Pain/discomfort**			
Low HL (ref. High HL)	**1.24 (1.01, 1.54)**	**1.23 (1.01, 1.50)**	1.13 (0.95, 1.33)
With CD (ref. without CD)	N/A	N/A	**2.56 (2.18, 3.00)**
**Anxiety/depression**			
Low HL (ref. High HL)	1.07 (0.83, 1.38)	1.07 (0.83, 1.38)	1.04 (0.82, 1.32)
With CD (ref. without CD)	N/A	N/A	**2.14 (1.76, 2.60)**

1: Log-binomial regression models, for the entire sample, model 1 adjusting for age, ethnicity, income, geographical location, model 2 further inclusion of education based on model 1, model 3 further inclusion of the presence of chronic disease based on the model 2. Significant PRs and 95% CI were shown in bold at *P* <0.05.

2: HL = health literacy CD = chronic disease.

The health literacy-stratified analyses (Table 3) showed that the adjusted PRs of having a chronic disease in the high health literacy group were larger (from 2.28 to 4.62) than those in the low health literacy group (from 1.99 to 3.87). The interaction of health literacy with chronic disease was significant for pain/discomfort impairments (*P* = 0.033), indicating PRs for pain/discomfort impairments related to chronic disease significantly differed by health literacy level. The interactions of health literacy with chronic disease were not significant for impairments in the other dimensions of EQ-5D. The analyses stratified by the presence/absence of chronic disease showed that the significant associations of low health literacy with more impairment in mobility and pain/discomfort were observed only in women without chronic disease, but not in women with chronic disease.

**Table 3:** **Adjusted** ^**1**^ **prevalence ratios (PRs) and 95% CI for impairments in EQ-5D, by HL** ^**2**^ **and CD** ^**2**^

	**HL level**		**The presence/absence of CD**		***P*** ^**3**^
	**High HL**	**Low HL**	**With CD**	**Without CD**	
	**With CD (ref. without CD)**	**With CD (ref. without CD)**	**Low HL (ref. high HL)**	**Low HL (ref. high HL)**	
**EQ-5D Impairments**	PRs (95% CI)	PRs (95%CI)	PRs (95% CI)	PRs (95% CI)	
Mobility	**3.86 (1.67, 8.95)**	**3.56 (2.00, 6.35)**	1.10 (0.58, 2.11)	**3.95 (1.47, 10.65)**	0.978
Self-care	**4.62 (1.90, 11.22)**	**3.87 (1.69, 8.87)**	0.79 (0.36, 1.73)	0.94 (0.25, 3.53)	0.797
Usual activities	**2.71 (1.81, 4.05)**	**2.46 (1.54, 3.92)**	0.80 (0.49, 1.31)	1.34 (0.70, 2.56)	0.928
Pain/discomfort	**3.19 (2.52, 4.05)**	**2.15 (1.76, 2.64)**	1.04 (0.87, 1.25)	**1.63 (1.13, 2.34)**	**0.033**
Anxiety/depression	**2.28 (1.73, 3.01)**	**1.99 (1.52, 2.61)**	1.00 (0.75, 1.34)	1.11 (0.73, 1.68)	0.791

1: Log-binomial regression models, adjusting for age, ethnicity, income, education, geographical location. Significant PRs and 95% CI were shown in bold at *P* <0.05.

2: HL = health literacy CD = chronic disease.

3: *P* for interaction by *wald χ* ^*2*^ tests between CD and HL in the log-binomial regression models for the entire sample. Significant *P* values were shown in bold at the level of less than 0.05.

Linear regression models predicting EQ VAS scores for the entire sample (Model 1 in Table 4) showed that the VAS score was lower in the low health literacy group (*β* = −3.27, *P* = 0.012), controlling for socio-demographics (except education), as compared with the high health literacy group. After further sequentially including education (Model 2 in Table 4) and chronic disease (Model 3 in Table 4), the parameters for health literacy were not statistically significant at the 0.05 level. Similarly, the presence of chronic disease (Model 3 in Table 4) was a significantly salient predictor of low VAS scores (*β* = −14.47, *P* < 0.001). The health literacy-stratified analyses (Table 5) showed that the high health literacy group (*β* = −15.48, *P* < 0.001) reported a somewhat larger decrement in the VAS scores related to the presence of chronic disease than the low health literacy group (*β* = −13.70, *P* < 0.001), though the difference was not statistically significant as indicated by the interaction of health literacy with chronic disease (*P* = 0.579). The analyses stratified by the presence/absence of chronic disease (Table 5) showed that the significant association of low health literacy with the decreased VAS scores was only observed in women without chronic disease, but not in women with chronic disease. Linear regression models predicting utility scores also showed the same results as those predicting EQ VAS scores for both the entire sample and stratified samples (Tables 4 and 5).

**Table 4:** **Multivariate linear regression models** ^**1**^ **predicting EQ VAS scores and utility scores for the entire sample**

	**Model 1**	**Model 2**	**Model 3**
	***β*** **(95% CI)**	***β*** **(95% CI)**	***β*** **(95% CI)**
**EQ VAS scores**			
Low HL^2^ (ref. high HL)	**−3.27 (−5.8, −0.73)**	−0.70 (−3.99, 2.60)	−2.57 (−5.34, 0.21)
With CD^2^ (ref. without CD)	N/A	N/A	**−14.47 (−16.73, −12.22)**
F value (*P*)	8.26 (*P* < 0.001)	6.24 (*P* < 0.001)	24.21 (*P* < 0.001)
R-square	0.05	0.05	0.20
Adjusted R-square	0.05	0.05	0.19
**Utility scores**			
Low HL^2^ (ref. high HL)	**−0.023 (−0.044, −0.002)**	−0.017 (−0.044, 0.009)	−0.017 (−0.041, 0.008)
With CD^2^ (ref. without CD)	N/A	N/A	**−0.117 (−0.137, −0.097)**
F value (*P*)	5.48 (*P* < 0.001)	3.83 (*P* < 0.001)	18.65 (*P* < 0.001)
R-square	0.03	0.03	0.16
Adjusted R-square	0.03	0.03	0.15

1: Linear regression models, for the entire sample, model 1 adjusting for age, ethnicity, income, geographical location; model 2 further adjusting for education based on model 1; model 3 further adjusting for the presence of chronic disease based on model 2. Significant regression coefficients (*β*) and 95% CI were shown in bold at *P* <0.05.

2: CD = chronic disease HL = health literacy.

**Table 5:** **Multivariate linear regression models** ^**1**^ **predicting EQ VAS scores and utility scores, by HL** ^**2**^ **and CD** ^**2**^

	**HL** ^**2**^ **level**		**The presence/absence of CD** ^**2**^		***P*** ^**3**^
	**High HL**	**Low HL**	**With CD**	**Without CD**	
**EQ VAS scores**					0.579
Low HL^2^ (ref. high HL)	N/A	N/A	−0.050 (−5.139, 5.039)	**−4.433 (−7.618, −1.247)**	
With CD^2^ (ref. without CD)	**−15.48 (−18.46, −12.50)**	**−13.7 (−17.08, −10.33)**	N/A	N/A	
F value (*P*)	15.38 (*P* < 0.001)	10.75 (*P* < 0.001)	2.539 (*P* < 0.05)	5.695 (*P* < 0.001)	
R-square	0.22	0.16	0.06	0.08	
Adjusted R-square	0.21	0.15	0.04	0.06	
**Utility scores**					0.647
Low HL^2^ (ref. high HL)	N/A	N/A	0.001 (−0.046, 0.048)	**−0.028 (−0.056, −0.001)**	
With CD^2^ (ref. without CD)	**−0.12 (−0.15, −0.09)**	**−0.11 (−0.14, −0.09)**	N/A	N/A	
F value (*P*)	10.30 (*P* < 0.001)	9.67 (*P* < 0.001)	1.80 (*P* < 0.05)	1.62 (*P* < 0.05)	
R-square	0.16	0.15	0.04	0.02	
Adjusted R-square	0.14	0.14	0.02	0.01	

1: Linear regression models, adjusting for age, ethnicity, income, education, geographical location (for brevity, we did not show regression coefficients for these covariates). Significant regression coefficients (*β*) and 95% CI were shown in bold at *P* <0.05.

2: HL = health literacy CD = chronic disease.

3: *P* for interaction by *t* tests between CD and HL in the linear regression models for the entire sample.

## Discussion

Our study examined the complex relationship between health literacy and HRQoL in the context of chronic disease. Low health literacy was associated with poorer HRQoL; health literacy emerged as a moderator of the association between chronic disease and HRQoL, and the association was weaker among low health literacy women.

We found that women with low health literacy had a higher risk of pain/discomfort impairments, even after adjusting for socio-demographics. The result was similar to previous studies [15,18-20]. For example, a study among U.S. elders showed that individuals with inadequate health literacy had lower physical and mental health scores as measured by SF-12 [15]. Another U.S. study found that inadequate health literacy was associated with more pain that interferes with normal work activities, besides poorer physical and mental health [18]. The data did not allow us to investigate the mechanisms through which health literacy affects HRQoL. However, past research provides three pathways that may associate low health literacy with worse HRQoL: 1) decreased access to and use of health care because of difficulty in navigating the health system, 2) increased stress burden related to the challenges of daily life, navigating the health system, and disease self-management, and 3) lower self-efficacy for incompetence to control over one’s life and surroundings [11]. More understanding of these pathways is needed if we are to develop cost-effective interventions to reduce health disparities among underserved and vulnerable populations with low health literacy. In addition, our finding—that adjusting for the presence of chronic disease eliminated the disparity in pain/discomfort impairments across health literacy groups—may help explain the widely observed poorer overall health status of low health literacy populations that is attributable to a higher prevalence of chronic disease among them [15,19].

Chronic disease strongly predicted impairments in all the EQ-5D dimensions; its association with pain/discomfort impairments varied by health literacy level and was less pronounced among women with low health literacy. These results confirmed our hypotheses that health literacy may modify the HRQoL responses to chronic disease, and that women with low health literacy perceive or report less subjective HRQoL impacts compared to those with high health literacy in accordance with Wilson and Cleary’s modified conceptual model of patient outcomes [5]. HRQoL is a subjective rating relative to a person’s life expectations, values, and social and cultural background. Thus, we might infer that the modification of health literacy in the HRQoL responses to chronic disease is attributable to the different threshold or expectation of women recognition or report the HRQoL impact by health literacy level [33]. Women with low health literacy have difficulty understanding or recognizing the signs or symptoms of chronic disease [34]. As a result, low health literacy may affect early presentation of symptoms (i.e., patients’ delay) or be associated with under-reporting of symptoms or HRQoL impairments [21,33]. Given that general health perceptions are among the best predictors of the use of health services [4], this under-reporting of HRQoL impacts is likely to inhibit individuals from seeking and sustaining care for chronic disease. Under-reporting of HRQoL impacts may also jeopardize physicians’ appropriate therapeutic decisions for chronic disease, because such decisions are based on the assumption that self-reported HRQoL impacts reflect a true representation of a patient’s experience or condition. Ultimately, this may lead to unmet health needs and widened health disparities among women with low health literacy.

Low health literacy women had lower EQ VAS scores and utility scores compared to those with high health literacy, even adjusting for socio-demographics (except education), indicating a negative effect of low health literacy on HRQoL. However, the inclusion of education eliminated the significant association of low health literacy with EQ VAS scores and utility scores. This could be explained by the evidence in previous research that the “overadjustment” of educational attainment may underestimate the effect of health literacy on health outcomes for educational attainment is highly correlated with health literacy [16,35]. A study among Korean community-dwelling elders found that the exclusion of education as a covariate increased the magnitude of differences in health status between low and high health literacy groups, especially in physical function and pain that interferes with normal work [19]. Thus, the hierarchical analyses in this study help better understand independent relationships between health literacy and health outcomes. The presence of chronic disease was also a strong predictor of low EQ VAS scores and utility scores; low health literacy women reported a slightly less decrement of EQ VAS scores and utility scores related to the presence of chronic disease than those with high health literacy. Essentially, the results of linear regressions for EQ VAS scores and utility scores suggested the tendency of health literacy as a moderator of the awareness and recognition of HRQoL impacts related to the presence of chronic disease.

In addition, the finding that the associations of high health literacy with less impairment in mobility and pain/discomfort and with higher EQ VAS scores and utility scores were not significant in those with chronic disease may be attributed to the low overall health literacy level among this subgroup. Inadequate responses to low health literacy level among women with chronic disease may result in poor health outcomes.

The health literacy issues must be explicitly addressed. This will require holistic interventions and policies that consider root causes of health disparities in medically underserved and vulnerable populations. Health literacy is viewed as “a clinical risk” in medical settings; in public health it is considered as “a personal asset to be built, as well as an outcome to health education and communication” [12]. Multifaceted and collaborative interventions to improve health literacy and help people develop competencies could result in increased individual control over health and the factors that shape health [12]. Interventions addressing on structural barriers from health care system are also warranted [36]. Healthcare professionals need to be aware of their patients’ limited health literacy. They should communicate in plain terms and use interview techniques so that patients with low health literacy are more responsive in medical encounters [34]. Simplifying and standardizing health-related information will lower literacy burden of materials and health related tasks, which can especially be beneficial to patients with low health literacy. Technological support, such as pictures, video, multimedia, and other decision aids, can facilitate communication about complex ideas. Medical planning then better meets the needs of patients, and creates a more humane and literate health society.

Self-reported HRQoL outcomes remain crucial in making healthcare decision and evaluating quality of care; thus it is needed to develop and validate the sensitive and efficient measurements among undertreated populations with low health literacy [9]. Further research is needed to explore health literacy as a potential moderator of self-reports among large and diverse samples. Our findings suggest that health literacy should be taken into account in the design and interpretation of the research on care-seeking behaviors, chronic disease, and HRQoL.

The study has several limitations. First, its cross-sectional design did not permit causal inferences. Further, both diagnosed chronic disease and HRQoL may be subject to reporting heterogeneity due to health literacy rather than true causal effect. While we can not be sure of the effect of such endogeneity issues, it is interesting to note that women with higher health literacy had a lower prevalence of chronic disease, and better HRQoL compared to women with lower health literacy. Second, data on chronic conditions were self-reported, which may result in biased prevalence estimates, particularly for those who are less educated and poor [28]. While we cannot be sure of the effect of education, it is interesting to note that the prevalence of chronic disease was much higher among less educated women. In addition, this pilot project focused on only a broad clinical factor of having a chronic disease, but did not address specific diseases because the sample was fairly young (mean age of 35 years), as the majority of the sample did not report having a chronic disease. Future research should go beyond the contribution of clinical disease, and examine how health literacy may modify subjective perception of the impact of chronic disease on HRQoL. Third, health literacy is measured using the revised Chinese Adult Health Literacy Questionnaire (R-CAHLQ) with a package of competencies for health. This may influence the way in which our study compares to previous studies, the majority of which measured functional health literacy alone. However, the definitions and measurements of health literacy are evolving and diverse across countries [37], with an increasing trend to measure multidimensional competences rather than a single competence [12,38]. Lastly, all participants came from one area and entirely consisted of younger women (i.e. mothers of students), resulting in sampling bias. A larger study with a representative sample from diverse locales could provide more generalisable implications for practice and policy.

## Conclusions

Health literacy modified the impacts of chronic disease on HRQoL, and low health literacy group reported less HRQoL impacts related to chronic disease. Research should address health literacy issues as well as root causes of health disparities for vulnerable populations.

## Abbreviations

HRQoL: Health-related quality of life
EQ-5D: European Quality of Life-5 Dimensions
R-CAHLQ: The revised Chinese Adult Health Literacy Questionnaire
PR: Prevalence ratio
CI: Confidence interval
CVIs: Content Validity Indexes
